# Crisis resolution and home treatment: stakeholders’ views on critical ingredients and implementation in England

**DOI:** 10.1186/s12888-017-1421-0

**Published:** 2017-07-17

**Authors:** Nicola Morant, Brynmor Lloyd-Evans, Danielle Lamb, Kate Fullarton, Eleanor Brown, Beth Paterson, Hannah Istead, Kathleen Kelly, David Hindle, Sarah Fahmy, Claire Henderson, Oliver Mason, Sonia Johnson, CORE Service User and Carer Working groups, Mary Clarke, Stephanie Driver, Pauline Edwards, Kath Fraser-Jackson, Jackie Hardy, Kate Holmes, Lyn Kent, Jacqui Lynskey, Brendan Macken, Mary Plant, Tony Rivett, Jo Shenton, Geoff Stone, Janice Skinner, Jules Tennick, Gen Wallace

**Affiliations:** 1 Division of Psychiatry, UCL, 149 Tottenham Court Road, London, W1T 7NF UK; 2 IMPACT SRC, Deakin University, Victoria, Australia; 3 Anna Freud National Centre for Children and Families, 12 Maresfield Gardens, London, NW3 5SU UK; 4 Oxford Health NHS Foundation Trust, Barnes Unit, John Radcliffe Hospital, Oxford, OX3 9DU UK; 5 Health Service and Population Research Department P029, King’s College London Institute of Psychiatry, Psychology and Neuroscience, De Crespigny Park, London, SE5 8AF UK; 6 School of Psychology, University of Surrey, Guildford, Surrey, GU2 7XH UK

**Keywords:** Crisis resolution teams, Home treatment teams, Acute care, Qualitative research, Mental health services, Implementation research, Service users, Carers, Severe mental illness

## Abstract

**Background:**

Crisis resolution teams (CRTs) can provide effective home-based treatment for acute mental health crises, although critical ingredients of the model have not been clearly identified, and implementation has been inconsistent. In order to inform development of a more highly specified CRT model that meets service users’ needs, this study used qualitative methods to investigate stakeholders’ experiences and views of CRTs, and what is important in good quality home-based crisis care.

**Method:**

Semi-structured interviews and focus groups were conducted with service users (*n* = 41), carers (*n* = 20) and practitioners (CRT staff, managers and referrers; *n* = 147, 26 focus groups, 9 interviews) in 10 mental health catchment areas in England, and with international CRT developers (*n* = 11). Data were analysed using thematic analysis.

**Results:**

Three domains salient to views about optimal care were identified. 1. The organisation of CRT care: Providing a rapid initial responses, and frequent home visits from the same staff were seen as central to good care, particularly by service users and carers. Being accessible, reliable, and having some flexibility were also valued. Negative experiences of some referral pathways, and particularly lack of staff continuity were identified as problematic. 2. The content of CRT work: Emotional support was at the centre of service users’ experiences. All stakeholder groups thought CRTs should involve the whole family, and offer a range of interventions. However, carers often feel excluded, and medication is often prioritised over other forms of support. 3. The role of CRTs within the care system: Gate-keeping admissions is seen as a key role for CRTs within the acute care system. Service users and carers report that recovery is quicker compared to in-patient care. Lack of knowledge and misunderstandings about CRTs among referrers are common. Overall, levels of stakeholder agreement about the critical ingredients of good crisis care were high, although aspects of this were not always seen as achievable.

**Conclusions:**

Stakeholders’ views about optimal CRT care suggest that staff continuity, carer involvement, and emotional and practical support should be prioritised in service improvements and more clearly specified CRT models.

**Electronic supplementary material:**

The online version of this article (doi:10.1186/s12888-017-1421-0) contains supplementary material, which is available to authorized users.

## Background

Crisis Resolution Teams (CRTs; sometimes known as Crisis Resolution and Home Treatment Teams) are specialist mental health teams providing rapid assessment and intensive home treatment for people experiencing a mental health crisis. They offer an alternative to unpopular and costly inpatient admissions [1, 2]. Pioneered in the USA and Australia, CRTs were mandated in England in 2000 [3] and have also been implemented nationally in Norway and Flanders (Belgium) [4]. Key underlying principles include assessing and addressing the social systems and environmental triggers and contexts within which crises occur [5]; enabling development of coping skills in these contexts, to avoid or reduce severity of future crises; and providing care in settings where there may be fewer power inequalities in relationships with service providers than in hospital settings [4]. Recommended characteristics include 24-h opening; direct access; rapid responses; multi-disciplinarity; a “gatekeeping” role for all potential hospital admissions; and a range of interventions including medication, practical help with daily living tasks, family/carer support, and interventions aimed at increasing resilience and preventing relapse [6].

Despite trial-based evidence that CRTs can reduce inpatient admissions and costs, and increase service user satisfaction with acute care [7–9], evidence of effectiveness and impact on inpatient admissions and bed use is less clear when scaled up to national level [10, 11]. Implementation in England and Norway has been highly variable, and service planners’ expectations and recommended characteristics have only been partly met [12–15]. A recent survey [14] found wide variations in provision of all key aspects of English CRT services. Almost no CRTs were fully implementing national policy guidance [6], and adherence to this guidance had not substantially increased since a previous survey in 2005/6 [15].

Although service users are generally more positive about home-based than hospital acute care, a number of stakeholder criticisms of CRTs have been reported, including problems with accessing services, poor intra-CRT and inter-service continuity of care, and a narrow focus on medication to the exclusion of desired social and practical support [16–19]. Qualitative work has identified features of CRT care that are valued by service users and/or experienced as problematic if they are absent or compromised. These include rapid and easy access, practical support, care provided by the same staff with opportunities to form supportive therapeutic relationships, and integration with other mental health services [16, 17, 20, 21]. However, these studies have typically been limited to a single stakeholder perspective and/or single sites [16], and little is known about carers’ views [4].

It is clear that CRTs in England function variably and often less than optimally, and may not meet service users’ needs. The CRT model has not been highly specified and evidence for its critical ingredients is scant [4, 16]. A more highly specified model with greater connection to stakeholders’ views of critical ingredients and optimal care could thus help to improve implementation, outcomes and experiences [22]. In order to inform this, the current study is a comprehensive multi-setting exploration of views on the critical ingredients of CRTs, and successes and failures in their implementation that triangulates the views of all main stakeholder groups (service users, providers, developers and carers).

## Methods

### Governance

The study was approved by the North West London Research Ethics Committee (REC ref. 10/HO722/84). In addition to a management group and an independent steering committee, the study was guided by three advisory groups comprised of service users (*n* = 13), carers (*n* = 7) and clinicians (*n* = 8), recruited from participating NHS Trusts or local or national service user research groups.

### Settings

With the exception of CRT developers, interviews and focus groups were conducted in 10 mental health National Health Service (NHS) Trusts in England, covering a range of metropolitan, mixed and rural areas.

### Participants

#### Service users and carers

We aimed to recruit service users and carers distributed evenly across the 10 locations, and broadly reflecting the overall CRT population in terms of the following demographic, clinical and service use variables: Gender, age and ethnicity, and for service users: diagnosis, number of previous CRT contacts, previous psychiatric in-patient admissions, and whether they had a mental health care co-ordinator. In addition to the above criteria for the person they cared for, carers were sampled according to their relationship to the service user, and whether or not they lived with the service user. Exclusion criteria were lack of capacity and insufficient knowledge of English for an interview. Potential service user participants were identified by CRT clinicians from consecutive client lists. Recruitment of carers was via service user participants, CRT clinicians and local carers’ groups. Interviews were conducted towards the end of, or in the 3 months following, CRT contact.

#### Practitioners

Practitioners were purposively sampled according to levels of seniority, professional experience and geographical area. Four groups of staff were included: CRT clinical staff; managers and those with clinical leadership roles in CRTs, senior managers within mental health Trusts; and those who refer to CRTs from primary and secondary care services. Data was collected via focus groups where feasible, otherwise via individual interviews.

#### CRT developers

In order to develop a broad perspective on the history and theoretical origins of CRTs, interviews were conducted with key experts involved in the development of CRTs internationally, including those involved in dissemination of the CRT model, research or producing written guidance about implementation. Respondents were identified from existing contacts and knowledge of the field of CRT development within the study team.

### Measures

Topic guides were developed in collaboration with project advisory groups, ensuring that priority issues and the concerns of each group were explored. They were tailored accordingly for each stakeholder group, but all covered: views on current CRT practice; access to and discharge from the CRT; aspects of CRT care seen as most important; interface with other related services; suggestions for good practice; and barriers and facilitators to achieving this. Areas of concern identified by previous research (e.g. continuity of care, types of care provided) were explored.

### Procedures

Written informed consent was obtained from all participants. The majority of interviews with service users and carers (79 and 90% respectively) were conducted by trained peer researchers (service users and carers) drawn from advisory groups; the others by non-peer study researchers. Interviews were audio-recorded and transcribed.

### Data analysis

Data were analysed using thematic analysis [23] within NVivo software. The analytic strategy combined inductive and deductive approaches. We explored aspects of CRT work perceived to be important, successful and unsuccessful, aiming to be sensitive to respondents’ concerns and experiences. Areas of convergence and divergence within and between stakeholder groups were explored. The large data corpus necessitated a staged approach: Using a small sub-sample of transcripts, an initial basic set of themes was developed to capture broad areas and organise data. This was progressively elaborated through an iterative process of reading and coding further transcripts, and group discussions. Later stages of analysis were focussed on developing a nuanced understanding of parts of the data corpus relating to our analytic aims. Members of project advisory groups representing all stakeholder groups, and including peer researchers who conducted interviews were involved throughout the analysis. These collective processes enhanced validity by encouraging high levels of reflexivity [24] and ensuring that the perspectives of stakeholder groups (service users, carers and practitioners) informed the analysis [25].

Further details of recruitment strategies, data collection and data analysis are provided in Additional file 1.

## Results

### Sample characteristics

Interviews were conducted with 20 carers and 42 service users (one of which was accidentally not recorded so excluded from qualitative analysis). Table 1 shows respondent characteristics that relate to our sampling criteria.

**Table 1:** Demographic and service use characteristics of service user and carer respondents

	Service users *N* = 42	*N* (%)	Carers *N* = 20	*N* (%)
Gender	Male	14 (33%)	Male	8 (40%)
	Female	28 (67%)	Female	12 (60%)
Age	16–24	5 (12%)	16–24	0
	25–34	11 (26%)	25–34	2 (10%)
	35–44	13 (31%)	35–44	3 (15%)
	45–54	8 (19%)	45–54	7 (35%)
	55–64	5 (12%)	55–64	5 (25%)
	65+	0	65+	3 (15%)
Ethnicity	White British	30 (71%)	White British	16 (80%)
	White Irish	1 (2%)	White Irish	1 (5%)
	White Other	2 (5%)	White Other	0
	Black Caribbean	3 (7%)	Black Caribbean	0
	Black African	1 (2%)	Black African	0
	Indian	1 (2%)	Indian	0
	Asian Other	2 (5%)	Asian Other	0
	Mixed White/Asian	0	Mixed White/Asian	1 (5%)
	Other mixed ethnicity	0	Other mixed ethnicity	1 (5%)
	Other ethnic group	2 (5%)	Other ethnic group	1 (5%)
Relationship to service user	N/A		Partner	11 (55%)
			Parent	9 (45%)
Lives with service user	N/A		Yes	15 (75%)
			No	5 (25%)
Number of times service user used a CRT	Once	17 (41%)	Once	10 (50%)
	2–5 times	18 (43%)	2–5 times	7 (35%)
	6–10 times	7 (17%)	6–10 times	2 (10%)
	> 10 times	0	> 10 times	1 (5%)
Most recent service user contact with CRT ended	Still receiving CRT support	7 (17%)	Still receiving CRT support	5 (25%)
	< 1 month ago	21 (50%)	< 1 month ago	6 (30%)
	1 to 3 months ago	10 (24%)	1 to 3 months ago	5 (23%)
	> 3 months ago	4 (10%)	> 3 months ago	4 (20%)
Previous service user in-patient admission	Yes	32 (76%)	Yes	16 (80%)
	No	10 (24%)	No	4 (20%)
Most recent hospital discharge	< 3 months ago	15 (36%)	< 3 months ago	5 (25%)
	3–12 months ago	9 (21%)	3–12 months ago	5 (25%)
	1–5 years ago	5 (12%)	1–5 years ago	3 (15%)
	> 5 years ago	3 (7%)	> 5 years ago	3 (15%)
	N/A	10 (24%)	N/A	4 (20%)
Service user has care co-ordinator	Yes	22 (52%)	Yes	11 (55%)
	No	14 (33%)	No	6 (30%)
	Not sure	6 (14%)	Not sure	3 (15%)
Diagnosis (reported by service user / carer)	Affective Disorder	21 (50%)	Affective Disorder	10 (50%)
	Psychotic Disorder	11 (26%)	Psychotic Disorder	5 (25%)
	Personality Disorder	3 (7%)	Personality Disorder	2 (10%)
	Unknown	7 (17%)	Unknown	3 (15%)

We conducted 26 focus groups and 9 individual interviews with a total of 147 practitioners. Focus groups were made up of 2–10 respondents (average of 4). Characteristics of these respondents are shown in Table 2. Eleven international leaders in CRT development were interviewed. These people had backgrounds in psychiatry (*n* = 5), nursing (*n* = 4), psychology (*n* = 1), and health service management (*n* = 1) and had worked in CRTs for 16.8 years on average. Six were based in England, with others located in Australia (*n* = 2), Holland (*n* = 2) and Norway (*n* = 1).

**Table 2:** Professional and demographic characteristics of professional respondents

	Subgroups	*N* (Total = 147)	Percent
Data collection	CRT staff (10 focus groups)	61	42%
	Senior staff (7 focus groups, 1 interview)	39	27%
	Referrer to CRTs (8 focus groups, 1 interview)	33	22%
	Senior managers (1 focus group, 7 interviews)	14	10%
Service	CRT	88	60%
	Community mental health team/equivalent	17	12%
	In-patient service	5	3%
	Senior management	15	10%
	Early intervention service	2	1%
	General practice	2	1%
	Liaison psychiatry	2	1%
	Other	3	2%
	Not specified	13	9%
Role	Nursing	45	31%
	Senior nursing	6	4%
	Team leader/manager	23	16%
	Senior NHS manager	16	11%
	Consultant psychiatrist	11	8%
	Other psychiatrist	9	6%
	Support time and recovery worker	11	8%
	Social worker	8	5%
	Occupational therapist/activity co-ordinator	4	3%
	G.P.	2	1%
	Psychologist/therapist	3	2%
	Pharmacist	1	1%
	Not specified	8	5%
Years worked in NHS: mean (s.d.) (*n* = 121)		15.8 (9.2)	
Years worked in CRT: mean (s.d.) (*n* = 99)		5.1 (3.5)	
Years at current service: mean (s.d.) (*n* = 116)		5.8 (4.4)	
Gender	Male	71	48%
	Female	75	51%
	Unknown	1	1%
Age group	16–24	2	1%
	25–34	26	18%
	35–44	42	29%
	44–54	54	37%
	55–64	14	10%
	65+	1	1%
	Unknown	8	5%
Ethnicity	White British	97	66%
	White Irish	4	3%
	White Other	5	3%
	Black Caribbean	5	3%
	Black African	6	4%
	Black Other	1	1%
	Indian	7	5%
	Pakistani	1	1%
	Asian Other	5	3%
	Mixed White / Black African	1	1%
	Mixed White / Asian	2	1%
	Other Mixed	1	1%
	Other ethnic group	4	3%
	Unknown	8	5%

### Qualitative findings

Eleven features of CRT work were identified as important or valued across all stakeholder groups. These were organised into three broad domains: i) organisation of care; ii) the content of CRT work; iii) the role of CRTs within the acute and continuing care systems. For each feature, similarities and variations in stakeholders’ views on successful and unsuccessful aspects of current CRT practice, and tensions in implementation are considered. At some points, numbers in stakeholder sub-groups for whom themes apply are provided, in order to give a flavour of their commonality and spread, and to indicate how stakeholder sub-groups may vary. Further details of results and additional illustrative quotes are contained in Additional file 2.

#### The organisation of CRT care

##### Accessibility and speed of response

Ease of access and speed of response in initial CRT contact were identified as important by all stakeholder groups. More than a third of service users and a quarter of carers described the sense of support rapid referral provided as the most helpful feature of their overall CRT experience.

*“One of the things that first strikes me is the availability and the immediacy of it…So the fact that the crisis team are so accessible at the point when you’re actually in crisis is just almost... it feels like a miracle at the time. I remember the first time it ever happened I couldn’t believe how sudden it was and how they could offer something that was so supportive in terms of an everyday contact. So I think that that really is the primary plus point about it”.* (SU28^1^)

At least a same-day response was advocated by service users and carers. CRT staff also stressed the importance of rapid responses, seeing service users in person soon after a referral and setting target response times (e.g. within 4 h of a referral), although the pressures this places on CRTs were acknowledged. For clients previously on the caseload, CRT staff and developers reported that self-referrals (not via health professionals) were usually appropriate, and users and carers with experience of this valued it highly. For clients not known to services, a majority view was that CRTs should assess and accept referrals 24/7. Practitioners’ comments reflected implementation tensions between maximising accessibility, and concerns about large volumes of potentially inappropriate self-referrals if no filtering process was in place.

Experiences of referrals from primary care were variable: there were some accounts of rapid and appropriate referrals that minimised distress, but some practitioners described large volumes of general practitioner (GP) referrals that did not meet CRT risk thresholds [see Additional file 2 for details]. There was a common view across stakeholders that hospital Accident and Emergency (A&E) departments were the least satisfactory referral pathway. Some service users and carers had experienced long waits in stressful and uncomfortable A&E environments. CRT staff criticised the quality of assessments and appropriateness of referrals from A&E and psychiatric liaison colleagues. However, their value in providing emergency assessments was recognised, especially at night, when other sources of help are unavailable.

*“I know it’s a bit of a broad brush statement, but there’s lot of referrals that take place in A&E where people have maybe had a bit too much to drink and have taken an overdose, the hospital are reluctant to discharge them without a psychiatric assessment, so the team go out or this one person goes to A&E, does an assessment, ends up saying actually you don’t need any follow up from psychiatric services.”* (Practitioner group 32, CRT referrers)

For both new referrals and existing clients, there was also agreement across all groups about the need for a 24/7 service offering at least rapid telephone access to clinical advice. Around a third of service users and carers described experiencing this as reassuring, although a small minority of both groups described negative impacts of being unable to successfully make phone contact, or feeling let down by a phone-only overnight service. Staff and CRT developers acknowledged that providing a more substantial 24/7 service has significant resource implications.

##### Regularity, reliability and clarity

Regular contact with CRT staff was extremely important for service users and carers, many of whom felt that visits daily or more frequently facilitated trust and emotional support (see section 2.2 below). Regular visits were cited as the most helpful aspect of treatment by a third of service users and a quarter of carers.

*I: What would you say was the most helpful thing about the crisis team?*

*SU07: It was the daily support, definitely; knowing that, when I got up each morning, if I was frightened or upset, I knew that someone was coming to see me.*

For the most part, services appeared to be successfully providing regular contact, although a few service users said that infrequent contact had been the least helpful aspect of their care. While practitioners also viewed regularity as important, this was more because it enabled them to monitor risk and clinical changes. Service users and carers also described reliability and clear communication as helpful to forming trusting therapeutic relationships, and a small minority of both groups described large negative impacts when staff were not reliable (e.g. they did not visit at agreed times, or failed or were slow to do things they had agreed to).

*“They were supposed to come here one day, and it was the first time he felt comfortable enough to be on his own with them, and I went to work, and I rang him, just to make sure he was up out of bed, and I think I rang him to see how it had gone, and they just hadn’t turned up.” … “I think the fact that they forgot to come to see him, really, he was… I think he was at the point where he was just getting to trust them, and thinking that they might be able to help him, and then for them not to come…”* (C10)

Similarly, clear communication from the CRT at every stage of care was important. Some service users and carers identified lack of clarity as the least helpful aspect of their CRT experience, and scope for improvements was identified across all groups. Information about the CRT’s remit and contact details at the start of care, medication, and written discharge plans at the end of care were areas where practice often fell short of ideals. However, the majority of service users and carers reported clear communication about the timing and staffing of home visits, and plans for ending CRT support. A small number of carers identified confidentiality as a barrier to clear communication between themselves, the CRT and the person they cared for.

##### Flexibility

Flexibility was an ideal advocated by all stakeholder groups, related to individualised care, choice and the nature of crisis work. CRT developers and practitioners discussed this for several aspects of CRT work, including referral criteria and work with other services. For service users and carers, flexibility was most important in relation to timing and location of help, forms of support and duration of CRT contact. Some service users particularly valued flexible visiting times and involvement in planning this. Flexibility in the modality and duration of CRT contact was both advocated and valued. Five service users who had previously been acute in-patients found the fixed and limited duration of CRT contact (up to a month) the least helpful aspect of their experience. Additional file 2 provides more details and examples.

##### Staff continuity

Service users and carers greatly valued the same staff visiting them over time, because of the opportunities this provided to develop therapeutic relationships, emotional support and trust. However, having large numbers of different staff visiting, and the negative impact of this on relationship formation was one of their principal complaints.

*“The least helpful thing, for me, was not knowing the person that’s coming through the door. It was hard for me to talk to a total stranger. It was almost like, I don’t really know why you’re here, because I don’t know you, I’m not going to talk to you, because of the person that I am… Unfamiliarity to me, it’s quite difficult ... when I’m in a bad situation. … When I’m at the point where I need the crisis team I prefer to have somebody who’s familiar that I can have that continuity with.”* (SU08)

Significant minorities of both carers and service users felt these problems were exacerbated by poor communication between staff, and described receiving inconsistent advice, or having to repeat their story or correct staff about their diagnosis or medication. Many service users and carers would have preferred a single allocated CRT worker or a smaller team of visiting staff. Only three service users had not minded a variety of CRT staff visiting them. These people described staff as up-to-date and well-informed, and were told who would be visiting next, thus ensuring support was as seamless as possible.

Practitioners and CRT developers were aware of the importance for service users and families of staff continuity and its impact on trust and therapeutic relationships, and many recognised the need to improve this. Several strategies to prioritise continuity within a shift-working system were discussed, including a key worker system or sub-teams linked to geographical areas. Suggestions to enhance continuity between staff included: good information gathering and sharing within the team; regular handovers, team meetings and supervision; good administrative and IT support; staff time to prepare for visits; and regular training to ensure a consistent team approach. However, a number of practitioners described organisational challenges and resource limitations as barriers to implementing strategies to improve staff continuity.

##### Staff mix and experience

Experienced and professional staff, who can manage and respond to people in crisis and communicate clearly, were important to service users and particularly carers, six of whom cited this as one of the most helpful aspects of their CRT contact. Carers often valued input from a skilled outsider.

*“Well, they’re responsive, but also they’re professional. The trouble with being a parent is you’re so emotionally involved, and I have no medical expertise. And also, to an outsider and a professional skilled outsider, the person who’s ill can say things, particularly negative things about the family that you can’t say to your parents.”* (C04)

While the mix of staff types was important to some service users, especially the availability of support workers, psychologists, social workers and occupational therapists, for others this was less important than being visited regularly. Views on “peer workers” with personal experience of mental health problems also varied: whilst some service users and carers valued talking to someone with experiential knowledge, others preferred to see a team member with more ‘traditional’ qualifications.

In comparison, there was more agreement across staff and CRT developers about the desirability of a multidisciplinary CRT team, with most CRT developers and several staff groups identifying this as one of the most important aspects of good crisis teams. Teams consisting of nurses and psychiatrists complemented with social workers, occupational therapists, and psychologists were seen as able to bring a range of perspectives into crisis assessments and responses, for example elements from social systems, family or cognitive therapies.

*“I think it’s good if a team is as multi-disciplinary as possible with OTs and with social workers. We used to have a psychologist who did a lot of family work and also supported the rest of the team in doing that. That post was cut unfortunately a few years ago, and as a result a lot of the family work we used to do has just disappeared because people don’t feel… they lost their skill and it just fell by the wayside”.* (Senior CRT clinicians focus group 35)

However, only a minority of CRT staff groups described their own teams as multidisciplinary. Some described practitioners other than nurses or psychiatrists as part of, or linked to the CRT, but often thought they had insufficient time allocated to CRT work. Practitioners often described current overall staffing levels as stretched or inadequate. A mix of staff on each shift across both disciplines and seniority was seen as important, for example nurses qualified to dispense medication, and experienced senior staff to conduct initial assessments, formulate risk and care plans, and take risk-related decisions, allowing less experienced staff to deliver care*.* Practitioners saw CRT-specific training as essential [see Additional file 2].

#### The content of CRT work

The second broad thematic domain related to what CRTs do, within which three factors were considered relevant to good crisis care.

##### Involving the whole family

Considering a mental health crisis within a family or social system was described as an ideal by practitioners, CRT developers and carers alike, although there were variations between stakeholder perspectives. Family/social systems were discussed most by CRT developers, who advocated mapping a person’s social networks at the point of assessment in order to guide CRT interventions.

*“Who are the key people involved in that service user who can help us understand what’s going on, and how can they help in managing this crisis? What’s the ramifications of this crisis and what do we… what are we going to need to fix him in the social context, what can we start with that now? I think they’re the good principles of it.”* (CRT developer 02)

In comparison, whole family involvement was discussed less by practitioners, who described initial home-based assessments informed by family members as valuable in developing a holistic view of the crisis, and deciding on the suitability of home treatment. Only a minority of practitioners and CRT developers thought CRTs should provide interventions to support the family’s needs, usually in the form of respite for primary carers, through extended visits or access to crisis beds. A more common view was that CRTs should signpost carers to support groups, counselling, family therapy, or advice on carers’ entitlements. Some practitioners acknowledged that carers’ views and support needs were often not considered adequately, and saw some barriers to family involvement, including the time-limited nature of CRT work, a focus on medication over social aspects of crises, lack of skills in social systems working, and resource limitations that impacted on length and frequency of visits, and duration of CRT care. Carers wanted to be involved in and informed of service users’ treatment and discharge decisions, but often described this not happening.

*“They didn’t listen to us as carers. We know the person we’re caring for.”* (C20)

Carers often felt their views had not been considered, for example, in deciding on CRT discharge dates, and most had not experienced CRT interventions involving the whole family or addressing social aspects of the crisis. However when specific carers’ support was provided, it was described positively. Carers valued having time with CRT staff without the service user, so they could relay any concerns privately, and being asked about their own wellbeing. Only a minority of service users spoke about family involvement, describing family members being involved only if they happened to be at home when CRT staff visited, although noting that this had been useful.

##### Emotional support

Provision of emotional support from CRT staff was extremely important for service users, the vast majority of whom reported receiving this to some degree, while many described this as the most helpful aspect of their CRT contact. Emotional support was also important for carers, some of whom described support for the service user as the most useful aspect of CRT contact, while others had received particularly valued emotional support themselves. A few service users and carers said they had not received any emotional support from the crisis team, feeling that they were not listened to or understood.

The majority of service users and carers attributed feeling emotionally supported to CRT staff spending time with them, and opportunities to tell their story and talk openly about their feelings and difficulties. These were often described as being facilitated by organisational features of care discussed above, particularly regular home visits and continuity of staff that allowed therapeutic relationships to be developed. Staff who listened, and were caring, helpful and friendly helped service users to feel understood, and less isolated and distressed. Service users particularly valued and felt reassured by staff who conveyed non-judgemental, non-stigmatising and normalising messages, and hope of recovery, and encouraged them to look forward and move on. Together with having sufficient time to talk, this helped service users feel valued and promoted trust. A smaller number of carers talked about similar issues, and how this gave them a sense of reassurance and respite. Both service users and carers valued having someone external to their lives they could talk openly with

*“Just listening as well, listening to you going on. And becoming somebody that you could trust, because you were telling them an awful lot about yourself and they’re not judging you. And like I say they never made you feel as though it was you being stupid, or they never made you feel little … you weren’t taking their time up and wasting their time.”* (SU22)

In comparison, emotional support and relationship development were discussed less by professionals, although they recognised that their personal style in relating to service users and families was important. CRT staff in particular identified excellent interpersonal skills as important to developing good relationships with clients and families. A combination of skills similar to those identified by service users and carers was identified: being caring, friendly, supportive, a good listener, respectful, non-judgemental and courteous. Factors that facilitated or impeded emotional support were discussed by all stakeholders. Among facilitating features, trust was central and was often related to features described in section 1 (rapid responses, staff continuity, reliability and regularity). Lack of staff continuity, brief visits, and a focus on medication were seen to compromise emotional support, although there were some reports that if previously unknown staff were friendly and well-informed by colleagues, these limitations could be overcome [see Additional file 2 for further details].

##### CRT interventions

Practitioners and CRT developers thought CRTs should provide a combination of medical, practical and psychological interventions, with some emphasising the importance of addressing social aspects of mental health crises. However, in practice, all stakeholder groups reported that medication was the principal intervention delivered, with variable amounts of practical support, and only minimal delivery of other interventions.

*Medication:* There was a common view that medication is central to CRT treatment. Many service users reported that CRTs prescribing new medication, or reviewing or modifying previously prescribed medication had been very valuable [see Additional file 2 for details]. However, a common concern across all stakeholder groups was that CRTs are often too narrowly focussed on medication, at the expense of other interventions, or time talking to service users.

*“They pop in for no more than 4 minutes to make them take their meds […] and every now and then ask a question. […] But it’s a pointless visit in my mind, because, other than delivering meds, how are you delivering care?”* (C03)

Some practitioners attributed this to lack of resources or time, so that staff sometimes delivered medication in very brief home visits. Some service users complained that CRT staff were unresponsive to concerns about medication side-effects, and a small number of carers found communication and organisation surrounding medication problematic.

*Practical and social interventions* were valued by many service users, although several said that needed practical support had not been provided. A quarter of carers said this had been the most helpful thing provided by the CRT. Examples of valued practical support included help with daily living activities (cooking, shopping, housework, personal hygiene, managing finances), transport, resuming social activities, and welfare or housing issues. Practitioners discussed these activities as vehicles for therapeutic relationship formation that were normalising, non-stigmatising and empowering, even when people were unwell.

*“I think a lot of the crisis work that we do is about empowering the individuals to take control of their own lives, rather than going into hospital where it’s all sorted out for them”.* (Practitioner group 20; senior staff)

Across all stakeholder groups there was a general view that the social or practical issues underlying a mental health crisis should be addressed by practical interventions provided by CRTs [see Additional file 2 for details]. Only a minority of practitioners felt it was not appropriate or feasible for CRTs to provide practical support given their time and resource limitations. Some were critical of a trend towards CRTs increasingly signposting and referring to other services, rather than providing support themselves.

*Other interventions:* Psychological input to CRT teams (assessing needs for further psychology input, guiding CRT staff in using principles from CBT or family therapy) was advocated across stakeholders. However, only a small number of service users reported receiving interventions such as cognitive-behavioural therapy (CBT) or solution-focused therapy. These were mainly valued, but a larger number said they had wanted but not received psychological input. Practitioners said that staffing and resource constraints meant that CRTs could usually only provide brief interventions targeting, for example, goal setting, sleep hygiene, problem solving, anxiety, medication adherence or suicidality. Respondents from all stakeholder groups advocated CRTs providing self-management support (skills-based interventions, such as mindfulness, relaxation techniques, coping strategies, and anxiety or anger management). Crisis plans and relapse prevention work were discussed by a small number of practitioners. Physical health checks were discussed by a minority of respondents. Some practitioners thought this was commonly neglected by CRTs, others described relying on health checks conducted in acute psychiatric wards and primary care. A small number of service users described the CRT encouraging or signposting them to address physical health problems.

#### Role of CRTs within the care system

This topic was discussed mainly by practitioners and CRT developers who identified the principal functions of CRTs as gate-keeping acute hospital admissions and providing a home-based alternative. However variations in interpretation and implementation of these roles, and the need for clearer definition and a shared vision of crisis services were consistently expressed.

##### Gate-keeping acute in-patient care

Most of the CRT developers identified this as the most important role for CRTs. The majority of practitioners thought that CRTs should assess all potential hospital admissions in person, and decide who receives CRT support. The extent to which this happened in reality varied considerably, and some respondents commented that absence of a gate-keeping role undermined the functioning of CRTs.

*“I think it impacts on their ability to be able to function because I think the majority of their time should be spent gatekeeping those people who might need inpatient care, and delivering home treatment. And the greatest proportion of the time should be delivering home treatment as a functional alternative to hospital admission.”* (CRT developer 04)

A physical presence on acute in-patient wards and at assessments was thought to be necessary for effective gate-keeping. Tensions between CRT staff and consultants and ward staff regarding admission decisions were identified as potential barriers.

##### Providing home-based treatment

Practitioners discussed the value of home treatment in allowing service users to maintain social contacts and daily routines, and be treated in the least restrictive environment. The majority of service users and carers saw similar advantages and thought recovery was more rapid at home.

*“I think that could be why I got better quicker, because I was in my familiar surroundings, and not locked away, not under a regime. […] It’s more relaxing at home, I can see my kids, I couldn’t see them in there, and that hurts. I think it helps in getting better quicker.”* (SU20)

Service users also valued greater privacy, feeling safer than in stressful acute in-patient wards, and more equal relationships with staff. A small number of carers commented that, at times, they would have preferred their family member to be hospitalised for their safety, but most still believed home-based care was preferable. A concern about whether home treatment was based on clinical need or a requirement to reduce and shorten hospital admissions was expressed across stakeholder groups. A few service users said that their preferences for admission were not considered, and attributed this to reduced availability of hospital beds. Some practitioners thought that a primary aim should be to extend choice rather than reduce admissions.

##### Continuity and communication with other services

Good relationships and communication with other services (especially acute wards and community mental health services), responsiveness to referrals, and clear referral criteria were described as important for effective CRT work by the majority of practitioners and developers. However, in practice, problems in continuity of care and effective inter-service communication were reported across all stakeholder groups. There was nearly universal experience among practitioners and developers of misunderstandings and lack of knowledge about CRTs amongst referrers, particularly GPs and A&E staff (see section 1.1).

*“Do community mental health teams really know what home treatment is, and what it can offer, and how it adds value? Do inpatient services really know what the difference is from an inpatient service, apart from the four walls around it? And what crisis home treatments do. I think that’s a huge issue for the services, and I would imagine it’s incredibly confusing for those out in primary care to know what on earth any of us are trying to do.”* (Practitioner focus group 23; senior managers)

Joint working at the point of CRT discharge was considered particularly important to ensuring continuity of care, for example informing GPs and other mental health services about medication changes, and ensuring physical health monitoring and interventions are coordinated. Again problems in inter-agency communication were reported across stakeholders (although not universally), associated with service gaps, delays in receiving community support, and confusion. A number of strategies were suggested to help facilitate or improve communication, continuity and working relationships with other services [see Additional file 2].

## Discussion

Recent reviews of acute care in England have reported negative user experiences of CRTs [12, 19]. However, our data suggests that, despite some negative experiences of care, most mental health service users and carers value the basic principle of crisis management at home. Nearly a third of service users could not identify anything specifically unhelpful about their CRT experience, and this compares favourably with acute in-patient care for which consistently high levels of user dissatisfaction have been reported [19, 26].

Our study finds many areas of convergence between stakeholder groups about important ingredients of good quality crisis care in home settings. This allows us to compare how English CRTs appear to have evolved since they were mandated in 2000 to the original therapeutic models unpinning the CRT concept. Whilst there was broad stakeholder agreement with these as ideals for good home-based crisis care, practitioners in particular seemed to view the broad biopsychosocial focus of the original model as difficult to deliver in practice, at least within a resource-constrained system. Our data suggest that many English CRTs now operate using a more biomedical approach, enacted in brief home visits focused on medication and risk management with less, and more variable, psychosocial input. Similarly, the original strong focus on social systems working [1, 5] is advocated as an ideal across stakeholders (especially CRT developers), but appears to have been diluted considerably in practice. When provided, service users and carers value practical and social forms of support from CRT staff, and inclusion of carers in care processes and decisions, both intrinsically, and because they provide opportunities for developing trusting relationships with CRT staff. This chimes with other research highlighting the centrality of therapeutic relationships in acute care [26, 27]. Finally, consistent with a recovery-focused approach [28], there was a strong theme, particularly among service users, regarding the value of forward-looking, hopeful and non-stigmatising support from CRT staff.

In addition, there was broad agreement that CRT care is effective and appropriate when it is easily accessible, and provides prompt responses and regular home visits; and when CRT practitioners are reliable, and there is sufficient staff continuity and time to develop relationships with service users and families. These findings parallel those of several other studies that have explored critical ingredients of CRT care including limited numbers of different staff visiting, good communication, continuity of care within the CRT and with other services, and adequate staffing and visit time [16]; avoiding an overly medical focus [19]; the importance of practical help and carer involvement [17, 20, 29–31] and integration with other mental health services [32]. As well as those discussed above, other commonly identified problematic features in our findings include poor interfaces with other service providers, leading to referrals to CRTs and post-CRT follow-on care that were delayed or considered inappropriate by CRT staff (especially referrals via A&E and primary care).

Despite many commonalities, some areas of stakeholder divergence emerged. Carers stood out in reporting that they felt excluded from care processes, and that their views were often not considered. In several areas, although practitioners and CRT developers shared service users’ and carers’ concerns, they did not attribute as much importance to them. This was the case for staff continuity and limiting numbers of different staff visiting, emotional support, regularity and reliability of visits, and clear communication. For service users and carers these service characteristics were important because they provided opportunities to develop trusting and supportive relationships with staff. Practitioners often described how ideals like staff continuity were compromised by organisational challenges or resource limitations, but sometimes failed to appreciate the negative impacts when continuity, reliability or sufficient time to talk were not delivered.

Our findings refine understanding of what specific aspects of staff contact are important and facilitate therapeutic relationship formation, by highlighting how service users and carers value the combination of interpersonal skills (non-judgemental listening, a caring attitude, providing emotional support) and professional skills (experience, reliability and clarity of information provision). Similar findings are reported in acute residential settings by Sweeney et al. [27] who describe this combination of attributes as “professionalism plus”.

### Strengths and limitations

This is the first study to provide an in-depth qualitative exploration of the successes and limitations of CRTs across a wide range of contexts (10 NHS Trusts in England), from the multiple perspective of all relevant stakeholders (service users, carers, practitioners, managers, referrers and international CRT developers). The large qualitative data corpus (107 data sources), and the range of demographic, clinical, service use and professional characteristics of samples promote confidence that common themes may reflect the views of these stakeholder groups more generally. However, our use of some numbers to indicate the comparative spread of certain themes and concerns between stakeholder groups should be treated with caution and considered in the light of sampling strategies. Additionally, our sample is likely to under-represent services users and carers who are less engaged with services. While we were unlikely to hear critical views of CRTs in the sample of CRT developers we interviewed, the large and broad range of referrers and senior managers included in the practitioner sample allowed ample opportunity to hear the views of those who were critical of or sceptical about CRTs and their functioning. High levels of stakeholder involvement in design, data collection and analysis processes (see DS1) ensure that the concerns of these groups have been explored and reported. However, limitations of space mean we have focussed in this paper on common themes and prevailing views within each stakeholder group.

### Clinical implications

A striking finding of this study is that the original social systems approach to home-based crisis care [4, 5] appears to have been diluted in practice in many English CRTs. Carers and service users in this study have low expectations of meaningful family inclusion, support or education from CRTs. Family involvement now appears to take the form of considering carers’ views at the point of assessment. This is valued by carers, but often doesn’t happen, resulting in them commonly feeling excluded and uninformed about crisis care. Similarly, despite practical support featuring in original CRT formulations [33], and the value service users and carers place on this, the extent to which CRT staff provide help with practical and social problems is variable. When provided by CRT staff (rather than other statutory or voluntary agencies), help with daily living activities, resuming social activities, or financial or housing issues can be a vehicle for therapeutic relationship formation that is central to service user and carer experiences. This suggests the need for clearer role definitions for CRT staff to ensure that, as much as possible within resource constraints, practical support and social systems working are retained as important ingredients of home-based crisis management.

The value placed on staff being both professional and humane suggests that this skill combination should be prioritised in staff recruitment and development, with a focus on maximising opportunities for contact time and relationship formation within the confines of brief service engagement. This echoes other calls for more humane values in acute mental health care [12, 19], and for compassion as central to mental health care and clinical care in general [34, 35]. Service planners should consider the importance of human aspects of crisis care, and the significant negative impact on users and carers of poor staff continuity. Practices adopted by some CRTs to minimise numbers of different staff visiting a service user, and maximise staff continuity and information sharing could be implemented more widely. Finally, referral processes between CRTs and other services within the acute and community mental health care systems were frequently identified as problematic, suggesting that attention should be paid to the functioning of these wider care system and the development of clear pathways within these that are tailored to service users’ needs.

### Research implications

Findings have been used to inform development of a more highly specified CRT model, and tools to assess fidelity to this model [22]. There is a need to develop, test and implement resources to enhance key features of CRT service delivery and organisation, including continuity of care, provision of social and practical support to service users and families, and the quantity and quality of staff contact. A multi-centre trial is currently testing a service improvement programme designed to increase CRT model fidelity and improve outcomes [36]. Findings highlight challenges to CRT implementation in contexts of stringent resource limitations and complex service configurations. Research focusing on what facilitates or impedes successful implementation of good CRT care in different contexts is needed.

## Conclusions

There has been considerable investment in CRTs in England, with some success in providing less restrictive home-based alternatives to hospital care for acute mental health crises. However, it is not clear that CRTs have nationwide achieved their goal of reducing admissions, even though they did so in small-scale studies with expert leadership [4, 10, 11]. Data in this study suggest that CRTs as now implemented often lack several of the ingredients seen as critical by early pioneers, notably a social systems approach and the intensity of contact. While some stakeholders in this study seemed rather fatalistic about these deficits, this may in fact prevent the model from achieving its intended outcomes. By shedding light on how CRTs across demographically diverse locations in England are perceived and experienced by multiple relevant stakeholder groups, this study makes a valuable contribution to developing a more highly specified model of home-based crisis care, grounded in stakeholders’ views of its critical ingredients. This is of international interest as an important component of acute mental health care.

## Additional files

Additional file 1: Contains additional methodological details. (DOCX 15 kb)

Additional file 2: Contains additional findings and illustrative data extracts. (DOCX 45 kb)

## Abbreviations

A&E: Accident and Emergency
CBT: Cognitive Behavioural Therapy
CRT: Crisis Resolution Team
GP: General practitioner
NHS: National Health Service

## Footnotes

1 SU = service user respondent; C = carer respondent.
