# Supplementary material for: Crisis resolution and home treatment: stakeholders’ views on critical ingredients and implementation in England
Source: BMC Psychiatry. 2017 Jul 17;17:254. doi: 10.1186/s12888-017-1421-0 (PMC5512942; doi:10.1186/s12888-017-1421-0)
Supplement: Supplementary file 1 — Contains additional methodological details. (DOCX 15 kb) [file 12888_2017_1421_MOESM1_ESM.docx]

**Morant et al.**

**Additional File 1: Methodological Details**

**Recruitment Strategies**

Potential service user participants were identified by CRT clinicians from consecutive client lists. Participants were contacted initially by clinicians, and if they expressed an interest, subsequently by study researchers. Recruitment of carers was via service user participants, CRT clinicians and local carers’ groups using the same strategies and inclusion criteria as for service users. Participant practitioners were identified and recruited via managers of participating CRTs and mental health Trusts.

**Data Collection**

Potential service user and carer participants were identified and first approached about participation by clinical staff at participating services. Researchers provided an information sheet about the study and obtained written informed consent from all participants. Peer researchers (service users and carers) who collected data received specialist training in qualitative interviewing, and were supported and supervised by study researchers and a public involvement coordinator, himself a service user researcher. Service user and carer respondents were given £20 to acknowledge their time. The majority were interviewed at home, with a smaller number interviewed in NHS or university locations. Practitioner focus groups were facilitated by two study researchers. Interviews with CRT developers were conducted by telephone when face-to-face contact was not possible. All interviews and focus groups were audio-recorded and transcribed. Electronic data were stored in password protected files on a secure network; paper forms were filed in locked cabinets in a locked room.

**Data Analysis**

In order to enhance validity, a collaborative approach to analysis was adopted. A small group of researchers conducted the bulk of data analysis and met regularly with two larger groups: i) a “study researcher” group including academic researchers and members of the relevant stakeholder groups (clinicians, service users and carers); ii) a “peer researcher” group, made up of 15 research-trained service users and carers, 11 of whom had conducted interviews. These groups actively contributed to analysis by reading transcripts, reviewing the developing coding frame, and highlighting omissions and areas of interest for further exploration.

The large volume of data (107 documents in total) necessitated a staged approach: Analysis began with a small sample of transcripts from across the stakeholder groups, from which an initial basic coding frame to capture broad areas and organise data was generated. The sensitivity, specificity and conceptual clarity of thematic codes were progressively refined through iterative processes of reading additional transcripts, team discussions, and discussions with the larger study and peer researcher groups. These detailed data-driven processes were complemented by consideration of the broad impressions of academic and peer researchers who conducted interviews and focus groups, ensuring that both detail and principal themes were captured. Once all the data had been coded in this way, we summarised the principal topics and issues within each main thematic area and held review meetings with the larger groups to decide on an analytic focus. Later stages of data exploration became progressively more focussed in order to develop a nuanced understanding of parts of the data corpus that related specifically to stakeholders’ views of current and good practice. We also explored and compared responses to key questions about most and least helpful aspects of CRT contact (service users and carers); and characteristics of good CRT care (practitioners and CRT developers). Throughout this process, both commonalities and variations were explored, with a particular focus on variations associated with stakeholder groups or sub-groups based on our sampling criteria. The findings presented represent a selective account of our overall analysis, focusing on principal themes and variations.

Although black and minority ethnic (BME) groups were well represented in the service user and carer samples (21% and 15% respectively), we remained concerned that interviews with a relatively small number of BME service users and carers (12 in total) may not have adequately captured key issues for these groups. In response, we: i) looked specifically at data collected from BME service users and carers at an early stage of analysis; ii) ran an additional focus group with BME service users after the main data collection.
