# Supplementary material for: Crisis resolution and home treatment: stakeholders’ views on critical ingredients and implementation in England
Source: BMC Psychiatry. 2017 Jul 17;17:254. doi: 10.1186/s12888-017-1421-0 (PMC5512942; doi:10.1186/s12888-017-1421-0)
Supplement: Supplementary file 2 — Contains additional findings and illustrative data extracts. (DOCX 45 kb) [file 12888_2017_1421_MOESM2_ESM.docx]

**Morant et al.**

**Additional file 2: Additional results and illustrative data extracts**

**1. The Organisation of CRT care**

**1.1 Accessibility and speed of response**

There were mixed views and experiences of direct referrals from primary care. While examples were given by all stakeholder groups of appropriate G.P. referrals that led to rapid crisis responses, G.P.’s lack of experience with mental health conditions was also a common theme. A small number of carer respondents reported surprise at discovering the CRT service and wishing they had known of it earlier, suggesting that knowledge and awareness of CRTs among some G.P.s may be low. Similar experiences were mentioned by some service users. Several staff groups commented that G.P.s may have a lower threshold for defining a mental health crisis and may therefore generate a large volume of inappropriate referrals. The tension between this and G.P.s having a responsibility to refer people they believe are at risk was noted.

Professional respondents offered examples of strategies that had successfully facilitated easy and rapid access to CRTs: clear dissemination to referrers of CRT contact details and referral criteria; accepting and acting on phone referrals rather than waiting for a written form; including details of how to access crisis help in routine mental health service appointment letters; and a “no wrong door policy”, where CRTs take responsibility for referring people on to other services if they decline to take them on for treatment.

| **Sub-theme** | **Illustrative data extracts** |
| --- | --- |
| Speed of response | *“The key element for me now, if he had a crisis tonight, all I want is to be able to pick up the phone and say please come now. And I don't want to have to wait an hour for somebody to call me back or go through three different call operators. I just want somebody to say do this and we'll come and see you.”* (C09*). |
| Accessibility | I*: What part of the service do you feel that service users and carers really value the most from the Crisis Team?*  Trust manager 01: *I think in a good team … it’s the access. You know I think that what they value is the fact that they have got a safety net and they know that any time of the day they can phone up and speak to someone and they’ll get through to someone. There might be a paging service, but the teams from up here are very good at getting back to someone. So within five minutes, if not instantly, they’ll be talking to someone.*  *“You just get frustrated at the whole process of…they seem to have rules and regulations that make it very difficult. They were very nice on the telephone, but their rules don’t make it very easy for carers to speak to them. The fact that she wouldn’t take my word that the police had found him, and could we bring him to the hospital? It was, no, we had to wait for the police to bring him back here, and then the police had to ring her, then there was a long process of the police talking to them on the telephone, and them ringing back, before we got an answer to say, yes, we could take him to the hospital, and that they would see him there, with one of the psychiatrists.”* (C10) |
| G.P. referrals | *“There’s no doubt that the sort of things that G.P.s think are crises, don’t look like crises to mental health teams. I’m all in favour of being helpful to G.P.s and accepting that when G.P.s think there’s a crisis, that there is a crisis of some kind; but it’s not often one that looks to me like it needs a hospital admission or a home treatment team*.” (CRT developer 09) |
| 24/7 access | *“Overall, what matters most? I would say the fact that you can make contact with the crisis team 24 hours, 24/7. I think that is really good because before all this problem I would never have dreamt that I could have contacted somebody, say, on a Sunday at maybe three o’clock in the morning and there’ll be somebody at the end of the phone.”* (SU01) |

* C = carer respondent; SU = service user respondent.

**1.2** **Regularity, reliability and clarity**

| **Sub-theme** | **Illustrative data extracts** |
| --- | --- |
| Regular contact | *“I think, having someone to talk to on a daily basis really”* (SU04)  *“Just that I knew that they were always coming to see me every day, knowing that somebody out there cares”* (SU01) |
| Reliability | I: *If you had to say what was the best thing about them what would you say?*  SU20: *“I think that they actually did what they said they were going to do, and they didn’t put me off. They didn’t say, we’re too busy to do this, they came when they said they were going to come, or let me know if they couldn’t turn up. Everything they said, they would do they did, and you can’t get better than that, can you?”*  *“they ask you what time you want them to come and then … they couldn’t turn up at all”* (SU06) |
| Clear communication | *“I think they could maybe have made things much clearer and plainer, could have saved me an awful lot of angst, and anxiety.”* (C12) |

**1.3 Flexibility**

While valuing clarity, CRT developers and practitioner groups cautioned that narrowly defined and rigid referral criteria could create barriers to providing help where needed. For example, while CRTs should primarily provide an alternative to hospital admission, several practitioner groups and CRT developers described accepting clients with a remit to prevent future admissions, or providing support or advice to other services for cases not accepted by a crisis team. This facilitated good relationships with other services. A flexible approach to crisis management brought some challenges, including predicting workloads and matching staff skills to individual needs and preferences (for example when service users requested to see, or not see, particular members of staff). Planning staffing and shift patterns based on previous patterns of contact and need was advocated to manage this. Resource limitations were cited by some staff as a barrier to providing flexible responses to individuals’ needs. Service users gave examples of flexibility they had valued, including providing medication and regular telephone contact while staying away from home, and regularly reviewed, rather than fixed discharge dates.

| **Sub-theme** | **Illustrative data extracts** |
| --- | --- |
| Flexible referral criteria | *“I don’t think you can be too prescriptive and say we only see people like this. Because that means that people, often who are becoming unwell, often there needs to be some pragmatism about if, you know somebody is in the early stages of a relapse in their mental health, then getting in at the earliest point is really important so that they don’t carry on that trajectory of needing hospital care. And I think the team needs to be close enough to spot that, so that they don’t say, oh well they don’t need to be in hospital now so therefore we’re not taking them.”* (CRT developer 02) |
| Flexible contact and discharge | *“We can’t praise them enough for the flexibility they gave because K found being away from [*local town*] to be a very good place for recovery and E, the head of the crisis team, agreed that she could go and live with my parents in London for a couple of weeks and, sort of, do phone-in connections with the crisis team. And she said that normally they wouldn’t do this, but this sounded like a good idea and was mutually beneficial.”* (C17)  IV: *Was that long enough, or not long enough, or how was it?*  SU13: *I think that was long enough. Having said that… well, the doctor did want to discharge me about ten days earlier than I was discharged, and I didn’t feel ready to be discharged, so he was happy to carry on seeing me for a little bit longer.*  IV: *So listened to your concerns and kept you for a bit?*  SU13: *Yes.* |
| Tensions between clear service criteria and a flexible individualised approach | *“Patients are saying that they would probably like to define their own recovery more, i.e. have more time with the Crisis Team, which again is not without risk if you’re trying to target people who otherwise would be in hospital.* *So there’s tension between those two statements. If you’re saying that the teams are... When you go back to that awful statement ‘fidelity to the model’, that in a way can be a barrier to teams doing work, because they will say, well, that the model says that we work with people who otherwise would be in hospital. So if you use that as a mantra then it’s a convenient way of saying, well, this person wouldn’t be in hospital and therefore we’re not going to work with them.”* (Senior Trust manager 01)  IV: *Let’s look at their help, and the help and involvement that you received. Was there anything that could have been improved?*  C06: *Yes, if they don’t follow too much textbooks, and followed their instincts, because they felt that they should continue really, but the textbooks said enough, that it has to be for so long, and that’s it. Every patient has so much time, you see. I think they should go, for each, to make it an individual need, according to the person, not according to a time chart. Because after all, this is a person.* |

**1.4 Staff continuity**

| **Sub-theme** | **Illustrative data extracts** |
| --- | --- |
| Large number of staff visiting prevents relationship formation | *“They do know him but they don’t really know any of his hobbies, any of the things he likes. I don’t know how they would know each person. John doesn’t feel, if you like, that he has bonded with any of them. If you could have one person out of a team that would come and that was your person, if you like. I think that is so important and when you have a variety of people coming through the door you can’t really bond with any of them can you? He can be sociable but there is no connection.”* (C07)  *“I think it's a good service, but I think the only Achilles heel in it is the fact that there are different people every time, so you never build up a rapport with CRT. You never identify yourself with the same person twice. So that's the only downfall with CRT is that if it had the same people on every day doing the same shifts, then it would be more reassuring to see the same face and you can build up a rapport, you can build up a bond.”* (SU12)  *“I think the people who complain most are the people who value having a therapeutic relationship with an individual, and that’s something that we hear a lot of complaints about, is that a different person comes round every time.”* (Practitioner group 11, CRT referrers) |
| Continuity between staff and repeating information | *“I really do think it’s important that there is a little bit of consistency on the people that we see. I understand that you can’t always see her, but I think if you go from one person to the next person to the next person, you kind of start looking at explaining it again and explaining it again.”* (C08)  *“I think I saw about eight or nine* [CRT staff], *which I thought, I found quite hard the first week, being honest, but after that I found it quite nice, because I found that I was boring just one person every day with the same thing, and so it was quite nice seeing a variety of different people. And they all knew the back story, so I didn’t have to repeat myself every day, because that’s what I was dreading, I was dreading having to see a new person. A new person meant having to tell the story all from the beginning, whereas they already knew, they’d all read up on me before they came through the door … So that was good, I thought that worked well. There’s nothing worse than having to repeat something so many times when you’re feeling that bad.”* (SU40) |
| Lack of consistency of advice | *“One of the major hindrances to effective treatment with a good outcome is, because the way the teams work, that different members of the team invariably will be going at all different times to see a particular client, which is a constant complaint of the client. Unless all members of the team have similar levels of skill with evaluating both risk and symptoms, or whatever word we use, subjective state of the person, you get a patchy treatment experience. Because you get pieces of good input for that client, the person themselves feels, people understand me, we know where we are. Lo and behold next morning someone different rings at the bell, that person doesn’t read the signs and symptoms, can’t talk about the same things, gives completely different advice.”* (Senior Trust manager, 07) |
| Strategies to improve staff continuity | *“A lot of the time people are in crisis, it means they might not see the same person twice ever, so I think it’s working out the shift systems. … Somebody who’s in crisis should have one key person. I don’t think… I think it’s impractical for that person to visit them every single time, but the shift coordinator should be allocating that resource in a way that that person does go out as much as possible so there is some degree of continuity. Some teams have got around it by having sub teams within their teams, so locality teams that cover a certain geographical area. They might have red team and blue team that will cover a sub locality within that team so they’re all working the same patch, which minimises a little bit, but it is the most difficult thing too, because the teams are working around the clock, seven days a week, it’s very difficult to manage. So I think it’s about the team having an understanding of everyone’s caseload and trying to have at least one person who keeps the button on that one person throughout the journey. And it can be managed in different ways. They can make telephone contact even if they’re not visiting all the time, but as long as the person feels that there’s one person making contact and the carers feel as though there’s one person making contact.”* (CRT developer 04) |

- 1. **Staff mix and experience**

Practitioners saw staff training as essential, especially when staff start CRT work, because of the risk environment. Suggested elements of training included social systems training, alcohol and drug misuse assessments, working with personality disorders, psychological perspectives, and practical aspects of the role (e.g. handovers and information sharing). It was suggested that, with appropriate training the professional background of individual staff members should not matter, and the same basic training for all team members might be beneficial. Several members of staff mentioned regular supervision sessions as vital to a well-functioning team and helping to foster a strong team culture.

| **Sub-theme** | **Illustrative data extracts** |
| --- | --- |
| Experienced staff | *“(T)o hear it from somebody who’s a professional person and somebody who’s seen this kind of thing a hundred times, somebody away from your family, just gives you a feeling of security. So that, and also the fact that my family knew I was getting proper help”* (SU23)  *“The first thing is that it should be well staffed. It doesn’t have to be multidisciplinary in the sense of many disciplines, but it certainly needs to have experienced mental health professionals, and they’re usually nurses. They make the best, it seems to me, because they’re more accepted by the other people that the team has to work with, especially the acute ward. The nurses that I’ve met who work best in crisis resolution teams are nurses who really do understand acute inpatient care.”* (CRT developer 01) |
| Peer workers | *“If I’m truthful, I’d rather listen to somebody that’s lived through the same situations as what I’ve gone through, because they’ll know how I’m feeling, what emotions I’ve had, the anger, all that sort of stuff. Whereas, probably a mental health professional has read about it and studied it, but never experienced it. That’s a key thing, when you’ve experienced something.”* (SU04)  *“Later on, as we went in to the service, some of them said, you know, I’ve been unwell myself. This is what I found helpful. And then you, sort of, think okay, I’m going to listen to this advice because it comes out of experience.”* (C17) |
| Multi-disciplinary mix | *“I suppose other issues are the multi-disciplinary nature of the working. In my experience, we did get a reasonable mixture of social work staff and nursing staff with occupational therapists to some extent. I think that some of the important factors there are not really about the particular training that those people have gone through, but sometimes it is about their own demographics and their own academic background. So social workers, in my experience, were much more willing to think out of the box.”* (CRT developer 09) |

**2. The content of CRT work**

**2.1 Involving the whole family**

| **Sub-theme** | **Illustrative data extracts** |
| --- | --- |
| What is desirable or ideal | *“The key thing I think teams should be doing is family interventions, family approaches. … Working with social systems, taking advantage of the absolute central point of crisis, which is you’re working with somebody who’s in their own social milieu, and recognising that it’s that environment that created the crisis. … It’s the opposite from the reductionist notion of taking an individual out of the crisis, sticking them into a hospital and just treating their symptoms and divorcing them from their social side.”* (CRT developer 04)  *“I think there’s another really key important bit which is about the engagement of carers, particularly when you’re trying to support people at home. And I think for a crisis and home treatment team to work well, there needs to be that really robust engagement around the people that are supporting the individual outside of the professional services.”* (Senior Trust manager 23)  *“I think that’s a very important thing to do in the crisis situation, to look at the whole situation, and the whole family has that problem as well, the children and so on. So maybe to meet people in their own home is doing something about how the patient [unclear] and listening to their situation in another way.”* (CRT developer 06) |
| Reports of CRT practice | “W*hat is not being allowed for in crisis management is that there’s not just one person who’s had a trauma, there are two.”* (C19)  *“It's not just my daughter that needed help, it didn't just affect my daughter, it affected the whole family. Not just nearest, but it affected her aunties because they were all concerned.”* (C01)  *“They would talk to M. [carer] about his music and they were engaged with our life to a certain degree. But they have a job to do and they have to stick to it, that's how I see it. I don't think that's a negative thing, but that's what I would say, you have a job to do and your job is to make sure that I'm taking medication and that I'm responding to the medication in some way.”* (SU16) |

**2.2 Emotional support**

A commonly mentioned facilitator of emotional support from CRT staff was establishing trust. Across all stakeholder groups, features described in section 1 (rapid response, regularity and reliability, and staff continuity) were seen as encouraging trust and emotional support, and were valued by service users and carers partly for this reason. Other factors perceived to help users feel emotionally supported were choice regarding types of CRT input, inclusion in decision-making, and the “human” skills of staff. Staff who demonstrated both interpersonal and professional skills were advocated by both service users and carers. A degree of informality, use of humour, offering some personal opinions or relevant self-disclosure, and willingness to discuss service users’ interests all helped promote a sense of caring and connection. Some service users and carers described variable experiences of emotional support depending on which staff member visited them. For these groups, the most commonly mentioned barrier to building emotionally supportive relationships was lack of staff continuity. Service users and carers described finding it difficult to have a large number of different people visiting, and frustrations around repeating their stories to multiple staff members, at a time when consistency was important. This was acknowledged as a problem by some CRT developers and staff. However, respondents across all stakeholder groups described how ensuring staff communicate well, and have up-to-date information, in addition to being friendly and caring can ensure emotional support even when there are different individuals visiting. Other features described by members of all stakeholder groups as hindering emotional support were a highly structured approach to visits, and an exclusive focus on medication.

| **Sub-theme** | **Illustrative data extracts** |
| --- | --- |
| Aspects of emotional support:  feeling valued, understanding, hope, normalising, reassurance. | *Because I think what people value is someone going to spend time with them. I think what they value, and it’s a basic human need, is being valued themselves. So if you pick the phone up and give somebody a call and somebody’s going to come round see you within an hour that makes you feel valued and you value that; it’s pretty basic. And I think it’s as simple as that really. So I think that, you know, there’s medication that kind of thing, but a lot of people don’t value that, because you can get medication from your GP.* (Trust manager 01)  *“I think them understanding how low I was. I think their experience, even if my family didn’t understand how low I was, understanding that they’ve worked with people that were just as low, and that they’ve seen those people get better, and knowing that they can see in my eyes just how, they can see day by day just how low I was feeling, and that ability to, you know, to try and draw me out of that”* (SU40)  *“… it’s just the reassurance that this is a normal thing. Mental illness happens. It’s not the end of the world. You’re going to get better. Because when you… there’s so much stigma involved in mental illness and depression and when you’re depressed, you don’t see that light or whatever getting better and just having that constant reassurance this is just temporary, you’re going to get better, you’re doing the right things. That’s been the most helpful.”* (C14) |
| Facilitators of emotional support | *You have to be able to get their confidence, build the rapport therapeutically that is what it is about, and once you get them into your confidence, they will be able to work with you. I think that is our main tool, because in the community you’re working with the carers and the service users. You don’t have control 24/7, like how it is in the ward, they have to be having that big faith and trust in you, and that will happen only if we start looking at their own ideas, and giving them all the information.* (Practitioner group 28, Senior CRT staff) |
| Barriers to emotional support | *I remember that for a few days I had the same person, which was actually very nice that they sent the same person because, as I told you, I felt like I needed to be with the same person, you know, I felt like I already told them. I know they pass the information to the other person, but still it is much more comfortable for me, and I’m sure for most other people, to have the same person every single day because you do want to trust them and you do want to… it’s like a little relationship, you develop a little relationship with them and you need this reassurance from the same person. I think it was a little bit, I don’t want to say disturbing, to see a different person every day or every other day, but I think I would be much more comfortable to see the same person*.(SU21) |

**2.3 CRT Interventions**

Specific processes relating to medication that were valued by service users included: information about medication and side effects, both in leaflets and discussions with staff; being consulted about their preferences for treatment; prompt access to medication to manage an immediate crisis (e.g. sleeping tablets or sedatives); support or reminders to take medication (dosette boxes or watching it being taken); providing a limited supply of medication, where necessary, to prevent accidental or deliberate overdose; and home deliveries of medication. Carers particularly valued home deliveries of medication as they didn’t have to go to multiple chemists to find the required products. Some service users and carers thought CRT staff respecting their choice to decline medication was important, and CRT staff and developers placed importance on continuing to work with people who declined medication.

Some practitioners commented that delivery of practical interventions should be done by all CRT staff (not just social workers), especially as some teams have limited or no social workers input. A fully staffed multi-disciplinary CRT team and flexible staffing were seen by practitioners as necessary to providing practical support. The additional focus group with black and ethnic minority (BME) service users (see Data Supplement 1) produced many thematic similarities with the main data corpus. One exception was to highlight the importance of spiritual needs and concerns in periods of mental health crisis for BME service users and carers.

Signposting to other agencies (for example, drug and alcohol services) was advocated by practitioners for both people assessed by the CRT but not taken on, and during and following CRT contact, for example when practical support tasks could not be completed before the end of CRT contact. Service users and carers welcomed information about statutory health services and other community organisations.

| **Sub-theme** | **Illustrative data extracts** |
| --- | --- |
| Medication | *“The most helpful thing to me is they come and deliver the tablets... because that is a real faff having to sort things out sometimes, and some chemists don’t have this and some chemists don’t have that. And the fact that they come each day to make sure that we are okay. […] The thing that worries me is they don’t seem to be totally on top of the medication they give out, if I’m honest. You have got to watch what is being delivered.”* (C07)  *“They just focus on giving you medication. They don’t focus on nothing to let you go and have fun, they just focus on put it to sleep, medication and all those things, which is not good for your health.”* (SU02).  *“I have no doubt from what some of my patients tell me that home treatment can degenerate to a situation where somebody rings up and says, you’re on the list for a visit, can’t say when I’m coming, stay where you are. The person sits around waiting for hours and somebody comes in in a rush and says: ‘Have you taken your medication? Can I see you take your medication? Can you guarantee your safety? Are you going to commit suicide? Are you having any suicidal thoughts?’ A sort of interrogation and then they’re out; the delivery of medication and monitoring of suicidal ideas are the two interventions that all home treatment teams will do, and in some ways they’re the most trivial.* (CRT developer 09) |
| Practical support | *“The first task when you first engage with someone is to make some sort of relationship with them, and saying ‘take the blue pill’ might help, but being able to help them in practical ways is a much more engaging thing to do. Plus, it reduces the chaos in their life.”* (CRT developer 11)  *“The most helpful thing, I think, if you’re stuck in a rural location, like I was, is to be able to help you with transport or moving house or, you know, make you feel like you can change your situation. Just by knowing there is help, that you’re not stranded in any way, that they can help you get from A to B and get to certain offices … somebody had been to my door and posted me a letter straightaway about benefits and a job, a printout from the job centre that might have been of interest, you know. Just little bits and pieces that, you know, can make you feel a bit more positive.”* (SU33)  *“People do go into crisis because they can’t get bills paid, sometimes it literally is the practicalities that will get that person out of that crisis”.* (Practitioner group 32; CRT referrers) |
| Signposting | *“The bureaucracy is a barrier and the level of work has increased; the resources haven’t. The workload’s increased, and therefore you cannot go grab a van, offer to do this or do this or do that, because our time is limited. So you have to do more signposting, whereas we didn’t signpost as much because we would do some of that work ourselves. And that could be called more efficient, but what it does lead you to do is move more towards a medical model, more towards being a bit more prescriptive about some things, I think. There’s less out-of-the-box thinking that goes on now than perhaps there used to be.”* (Practitioner group 13; CRT staff) |
| Psychological interventions | *“I think most helpful has been seeing just one nurse once a week doing some CBT stuff. And I guess it is also helpful knowing that’s a point to check in if anything does go wrong.”* (SU34)  *“The thing I think I could have had quicker, and I still haven’t had is some type of therapy”.* (SU16) |

**3. Role of CRTs within the care system**

**3.1. Gate-keeping acute in-patient care**

| **Sub-theme** | **Illustrative data extracts** |
| --- | --- |
| Need for clearer definition | *“I think even the gate keeping role is confused now, because in terms of averted admission, some people say it’s about* preventing *that admission, and it’s that word, I guess, that needs to be quite precise and quite defined, really, as to what we do. Are we looking at working with acutely unwell people, or people that are moderately unwell, possibly bordering acute if they’re not.”* (Practitioner group 27; CRT staff)  *“It should be clear in the operational policy and that should be backed up by all managers, our leaders, our managers, and that information should be clearly communicated with all the community mental health teams and other healthcare professionals that we come into contact with so that we're all singing from the same hymn sheet.”* (Practitioner group 24; CRT staff) |
| Lack of gate-keeping role undermines functioning of CRTs | *“I think, every time we’ve seen gatekeeping taken away, it’s led to a slow deterioration in the service.”* (CRT developer 11)  *“A real key thing is the need to gate-keep hospital admissions. I think they need to sign up to that there is an ability to offer an alternative, less restrictive alternative to hospital admission where practicable and safe to do so. And to do that CRT staff need to be involved at every assessment at that level with somebody who’s likely to go into hospital.”* (CRT developer 02) |
| Value of CRT involvement in assessments | *“When I say gatekeeping, that should be face-to-face gatekeeping, and it should include all Mental Health Act assessments. “*(CRT developer 05) |

**3.2 Providing home-based treatment**

| **Sub-theme** | **Illustrative data extracts** |
| --- | --- |
| Maintaining social contacts and routines | *“It means many things to people and that's often about being able to maintain their social identity and their social credibility often as well, that they can carry on with their family, at home ... and they value that. Sometimes if somebody's got a housing problem, gets admitted to hospital and the housing problem is still likely to be there when they're discharged. If you’re treated at home, you’re tackling that, you’re looking at those things straight away.”* (CRT developer 02) |
| More rapid recovery from crisis at home | *“That he recovered much quicker because he was not in the hospital environment, and [unclear] he was in hospital, he was very easily affected by other patients, and it was a very negative effect on him. And he just recovered quicker, he was getting better quicker, and they did not need to overmedicate him, to keep him completely silent.”* (C06)  *“He gets better quicker at home than he does in hospital. With our scenario it’s a lot of anxiety and fear-based and stressed and he hasn't got that at home, even though it's stressful for us, we can keep the norm and it's peaceful, it's quiet. I can have a structure and a little routine, and he gets well a lot quicker.” (C18)* |
| Safety | *“In my home I feel safe. I feel I can be myself. For me, it’s the familiarity of being in this environment and knowing I am safe here”* (SU08) |
| Privacy | *“Well, I think you've got your privacy. Hospital is all right, really, but I don't want to go to hospital. I think if you are at home you've got your own surroundings. You've got your own food, your own television, your own private home, your privacy. I think the main thing is your privacy”.* (SU14) |

**3.3 Continuity and communication with other services**

A number of strategies were suggested to help facilitate or improve communication, continuity and working relationships with other services. These included: link workers who could liaise with community services, A&E departments, acute wards, the police and other mental health agencies; split posts across CRTs and acute wards, especially for senior psychiatrists; community staff, particularly care co-ordinators staying involved during CRT contact; proactive outreach to educate other services; raising awareness of CRT’s role and referral criteria; publicising service contact details and referral routes. Better education of GPs in both CRTs and mental health awareness was also recommended by some service users and carers.

| **Sub-theme** | **Illustrative data extracts** |
| --- | --- |
| Education / awareness raising for referrers | *“They were amazing, and it’s just a shame I think that people don’t know about them, the GPs don’t tell you. If you’re sitting in a waiting room there should be more posters and stuff, or your GP should be giving you that information rather than saying call the Samaritans or wait for an appointment to come through from the mental health team.”* (SU22)  *“An effective team does a lot of active outreach in work as well. So I think that one of the criticisms which is absolutely valid is that specialist teams can use their own specialism to confuse referrers and patients and the staff. So I like the teams here to be outreaching onto the wards, for example. […] The onus is on us to go and tell people what we do, as opposed to sit back and wait for other people to refer.”* (Practitioner interview 1; senior manager) |
| Staff continuity during crisis care | *“I probably would still recommend if possible that all consultant psychiatrists understand how crisis resolution teams work. And if they have patients that are being looked after by a crisis resolution team, then they keep in touch with that. They don’t hand over. There’s a fragmented way of caring for people that seems to be going on at the moment for various reasons and that doesn’t seem to me to be a very good arrangement either for patients or for staff.”* (CRT developer 01)  *“Support from the community mental health teams, not them just handing their patients to us and stepping out, but joining in with us.”* (Practitioner group 22; CRT staff) |
